# The inhibitory potential of *Zataria multiflora* and *Syzygium aromaticum* essential oil on growth and aflatoxin production by *Aspergillus flavus* in culture media and Iranian white cheese

**DOI:** 10.1002/fsn3.557

**Published:** 2017-12-15

**Authors:** Marzieh Moosavi‐Nasab, Jalal Jamalian, Hana Heshmati, Soroush Haghighi‐Manesh

**Affiliations:** ^1^ Department of Food Science and Technology School of Agriculture Shiraz University Shiraz Iran; ^2^ Seafood Processing Research Group School of Agriculture Shiraz University Shiraz Iran; ^3^ Department of Food Science and Technology School of Agriculture Tarbiat Modares University Tehran Iran

**Keywords:** Aflatoxin B_1_, antifungal compounds, inhibitory potential, *Syzygium aromaticum*, *Zataria multiflora*

## Abstract

Antifungal activity of essential oils (EOs) of *Zataria multiflora* (thyme) and *Syzygium aromaticum* (clove) against *Aspergillus flavus* growth and aflatoxin B_1_ production was studied in potato dextrose agar (PDA) and potato dextrose broth (PDB), as well as in Iranian white cheese as a food model. The results showed that the inhibitory potential of EOs in the PDB medium is more than PDA medium. Clove EO on PDB medium prevented fungal growth and aflatoxin B_1_ production at 300 and 100 ppm, respectively. However, the thyme EO was not able to inhibit fungal growth completely and showed the strongest inhibition effect at 400 ppm. EOs also had more inhibitory activity in laboratory culture media than the food environments. EOs in all concentrations reduced aflatoxin B_1_ production and fungal growth in cheese, but only the clove EO at 150 ppm was able to prevent the fungal growth and aflatoxin B_1_ production completely. Thyme EO reduced aflatoxin B_1_ value to below detection threshold (2 ppb) at 10 μl. Our findings propose EOs as a natural inhibitor to control fungal contamination of foodstuffs such as Iranian white cheese.

## 1 INTRODUCTION

Fungi are significant spoilage microorganism of foodstuffs during the storage, rendering them unfit for human consumption by retarding the quality of food. These fungi may produce as secondary metabolites a diverse group of chemical substances known as mycotoxins. Aflatoxins are the most hazardous mycotoxins that contaminate foods. They are produced by fungi such as *Aspergillus flavus* and *Aspergillus parasiticus* (*A. flavus* belonged to *Aspergillus* family). The favorable humidity and temperature for its activity are 80–90% and 36–38°C, respectively (Benavides, Villalobos‐Carvajal, & Reyes, 2012; Kamkar, Karim, Aliabadi, & Khaksar, 2008; Nazzaro, Fratianni, De Martino, Coppola, & De Feo, 2013).

Wheat (*Tricitum aestivum*), one of the most important staple foods used in Iran, is susceptible to fungal attack either in the field or during storage, and adversely affect quality of it that destined for human and livestock. Moreover, mold growth on cheese is a common problem for the cheese manufacturer during ripening and curing as well as for the retailer and consumer during refrigerated storage (Balaguer, Lopez‐Carballo, Catala, Gavara, & Hernandez‐Munoz, 2013; de Elguea‐Culebras et al., 2016; Reis et al., 2012).

Various chemical and physical methods have been studied for inhibiting the growth of fungi; among them, plant essential oils are known as one of the most effective and natural methods. Plant‐derived spices have been used in foods as flavoring agents. Nevertheless, there are a number of studies that demonstrated these compounds also exhibit antimicrobial activity by interfering and destabilizing the operation of the phospholipids bilayer of the cell membrane, enzyme systems, and genetic material of bacteria (Azizkhani, Misaghi, Basti, Gandomi, & Hosseini, 2013; Kim, Marshall, & Wei, 1995; Zhang & Lokeshwar, 2012). Examples of these antimicrobial spices are garlic, onion, cinnamon, mustard, black pepper, oregano, rosemary, Jamaican pepper, cardamom, and clove (Aguilar‐González, Palou, & López‐Malo, 2015; Li, Shi, Liang, Huang, & Chen, 2014; Manso, Cacho‐Nerin, Becerril, & Nerín, 2013; Ye, Dai, & Hu, 2013).

Thyme is a spice plant belonging to the Lamiacea family that geographically grows only in Iran, Pakistan, and Afghanistan. It is known as “Avishan‐e‐Shirazi” in Iran. Carvacrol and thymol are known as main phenolic compounds in its essential oil (EO) composition (Azizkhani et al., 2013). Clove is small shrub with evergreen and sharp leaves belonging to Myrtaceae family that is native to Indonesia and East Pacific. Eugenol is known as the main phenolic compound in its EO composition which contains 70–90% EO (Nikolić et al., 2015; Oussalah, Caillet, Saucier, & Lacroix, 2007).

In this study the inhibitory potential of clove and thyme EOs on growth and aflatoxin B_1_ formation by *A. flavus* ATCC 15546 was evaluated in culture media (potato dextrose agar [PDA] and potato dextrose broth [PDB]) and in Iranian ultrafiltered cheese.

## 2 MATERIAL AND METHOD

### 2.1 Preparation of EOs

Using industrial boilers, EOs of thyme leaves was extracted by hydrodistillation method in which the EO extraction efficiency was equal to 1.1%. The extraction efficiency for thyme has been previously reported as 1.66% (Moosavy et al., 2008), which was generally influenced by many factors, such as the plant leaves to water ratio, time, and temperature of extraction process. Initially, thyme was sun dried and the EO was extracted using hydrodistillation method in semi‐industrial boilers (manufactured by Kashan‐Iran Taghtiran Co.). For extraction of thyme EO, the proportion of water to dry leaves of *Zataria multiflora* was 800/100 (l/kg). Furthermore, the EO of clove was purchased from a Shargh Osareh Co. (Chenaran Industrial Estate, Mashhad, Iran). Oil extraction efficiency (weight basis) was calculated from the following equation (Shukla, Kumar, Singh, & Dubey, 2009):

$$\text{EO extraction percentage}\;\;\left(\%\right)=\frac{A-B}{A}\times 100$$

where *A* = weight of dry plant, *B* = weight of essential oil obtained.

For preparing the EOs for experiment, 0.5 ml of the desired EOs was mixed with 9.5 ml of distilled water and three drops of Tween 80 solution. Using a shaker device the reaction mixture was vibrated five times, each time for 30 s.

### 2.2 Standards

Standard for aflatoxin B_1_ were obtained from Sigma Chemical Co. (St. Louis, MO, USA). Stock solutions and standards were prepared and assayed according to modified AOAC Method 971.22 (Nesheim, Trucksess, & Page, 1998).

### 2.3 Preparation of conidial suspension

*Aspergillus flavus* (obtained from the Department of Plant Protection, Faculty of Agriculture, University of Shiraz, Shiraz, Iran) was cultured on PDA slope (Merck, Darmstadt, Germany) for 7 days at 28 ± 1°C. Conidia were cultured by adding 0.05% Tween 80 solution (Merck, Darmstadt, Germany) to the culture medium and gently scraping the mycelia with a sterile inoculating loop to release spores. Conidial concentration was determined by a hemocytometer and the suspension was diluted with 0.05% Tween 80 solution to give a final concentration of 10^6^ ml (Selvi, Joseph, & Jayaprakasha, 2003).

### 2.4 Measurement of the EOs effectiveness on the *A. flavus* growth in PDA culture media

Plates containing any of thyme or clove EOs were prepared. In order to culture *A. flavus*, one of the fungus 3‐day cultures was divided into parts (2 × 2 mm), and each of these parts was placed at the center of the plates and incubated at 28°C. The positive control plate contained fungi with no EO, and negative control plate only contain EOs with no fungi. The plates were checked every day, and the growth of fungus was enumerated as a function of hyphae growth radius. Comparison of this radius in plates containing different concentrations of EO and positive control plate was introduced as inhibitory effect of EO on the fungi growth (Gandomi et al., 2009).

$$\text{Inhibition percentage}\;\left(\%\right)=\frac{R0-R1}{R0}\times 100$$

where *R*0: radius of fungal hyphae growth in the positive control sample, *R*1: radius of fungal hyphae growth in the test sample.

### 2.5 Measurement of EOs inhibitory effects on the *A. flavus* growth in PDB medium

PDB medium was prepared in accordance with Difco method (Difco Laboratories, 1984). Erlenmeyer flasks containing a 3‐day culture of fungus were kept in incubator at 28°C for 10 days, and their contents were evacuated in preweighed filter paper that had been put in a desiccator. After complete drawl of fluid, filter paper containing fungal hyphae were transferred to 40°C vacuum oven. Then, the initial weight of filter paper was subtracted from filter paper containing fungus, and dry weight of fungus hyphae was found. Comparison of the hyphae weight of the test sample with the weight of fungus in positive control sample indicated the effect of the tested EOs in inhibiting the fungal growth. After separating the solid part, the permeate was excluded for measuring aflatoxin B_1_ tests (Rasooli et al., 2008).

### 2.6 Determining minimal inhibitory concentration and minimum fungicidal concentration

Suspension spore (0.1 ml) was added to each culture media containing different concentrations of EO. Plates were then incubated at 28°C for 48 hr. The lowest EO concentration which prevented visible microbial growth in that plate was determined as MIC or minimal inhibitory concentration of EO. Some agar pieces were removed from the plates containing MIC of EO and some agar pieces were removed from two plates with higher and lower EO concentration than MIC and finally they were cultured on PDA medium. Moreover, the first concentration at which visible microbial growth was observed was determined as the MFC or minimum fungicidal concentration (Mahboubi & Haghi, 2008).

### 2.7 Extraction of aflatoxin B_1_ from the samples cultured on solid media

Plates containing *A. flavus* in PDA medium after 5 days of incubation at 28°C were prepared for measuring aflatoxin B_1_ test. Culture medium content was completely transferred to 100 ml beaker and 8 ml of chloroform–water mixture with ratio of 1–15 were added to the beakers, and beakers’ content was mixed for 20 min. Then the reaction mixture was filtered by filter paper and permeated into second series of beakers. The remaining solids were returned to first series of the beakers, and all steps were repeated with 7 ml of solvent mixture. Finally, the filter paper was washed with 1–1.5 ml of chloroform, and all permeate obtained were collected in the second series of the beakers. Beakers were completely dried and washed with 3 ml of methanol and the collected solutions were poured in a special container with a lid that prevented the evaporation of methanol (Mayer, Färber, & Geisen, 2003).

### 2.8 Extraction of aflatoxin B_1_ from the samples cultured in PDA medium

First, 15 ml of filtered permeate and 15 ml of water and chloroform mixture were mixed for 20 min, passed through a separator funnel to separate the layers. Then, the lower layer, which contained aflatoxin B_1_, was collected in a 100‐ml beaker and completely dried in vacuum oven at 40°C. Beakers were completely dried and washed with 3 ml of methanol and the collected solutions were poured in a special container with a lid that prevented the evaporation of methanol (Razzaghi‐Abyaneh et al., 2008).

### 2.9 Measurement of aflatoxin B_1_ in food samples

In this study, high‐performance liquid chromatography was used to determine the level of aflatoxin B_1_ in each treated samples of EOs in PDA and PDB medium. For this purpose, 50 μl of condensed sample and various dilutions of the standard solution were spot on the chromatography plates coated with silica (G‐HR type, 20 × 20 cm and unsaturated with a thickness of 25 mm). The solvent system composed of ether, methanol, and water with volume ratios of 96, 3, and 1, respectively, were used to separate all four components of aflatoxin B_1_. The solvent system was poured in the tank and the door was closed. After development of the plates to 1.5 cm from the edge of top of the plate, chromatography plates were removed from the tank, and dried under the hood. To confirm the presence of aflatoxin B_1_, trifluoroacetic acid was sprayed on the plates. The spots were examined under ultraviolet light and their exact location was marked with a needle. The marked locations were completely scraped and transferred to test tubes. To each tube, 2 ml of methanol was added and kept in the refrigerator. The tubes were then removed from the refrigerator and after mixing for 5 min they were centrifuge at 330*g*. The aflatoxin B_1_ content of spots which was dissolved in methanol (supernatant phase) was carefully collected by micropipette and transferred to small containers and their absorption at 365 nm was read and compared with the standard curve to detect the amount of aflatoxin B_1_ content.

### 2.10 Estimation of *A. flavus* growth in Iranian white cheese

For estimation of *A. flavus* growth, using a homogenizer, 20 g of cheese samples were mixed with 90 ml of distilled water for 30 s. A serial dilution of the sample were prepared and cultured in a PDA media for 3 days of incubation at 28°C and the number of colony‐forming units (<150 cfu) were enumerated and multiplied by its dilatation rate to calculate the number of colonies per gram of sample.

### 2.11 Assessment of aflatoxin B_1_ production in Iranian white cheese

Measurement of aflatoxin B_1_ amount in cheese samples was performed using a high‐performance liquid chromatography devise (Agilent, USA) which was equipped with fluorescence detector and immunoaffinity type column. For each subsample, 2 g of the cheese sample was weighed into an acid‐washed pyrex flask at room temperature and extracted with 100 ml methanol:water (80:20, v/v) containing 5 g of sodium chloride by blending vigorously for 1 min in Waring Blender at high speed. The extract was filtered through fluted filter paper (24 cm, Vicam, Watertown, MA, USA) and 10 ml of the filtrate was diluted into 50 ml with deionized water and mixed vigorously. The mixture was passed through microfiber filter paper (11 cm, Vicam, Watertown, MA, USA) and 20 ml of filtrate was loaded on the immunoaffinity column (Aflatest, Vicam, Watertown, MA, USA). The column was washed two times with 10 ml of deionized water and the aflatoxins were eluted by passing 1 ml of HPLC grade methanol through column at a rate of 1–2 drop/second into acid‐washed HPLC vials. The eluate was evaporated to dryness under a flow of nitrogen at room temperature. The standards and samples were protected from direct light during all procedures (Kamkar et al., 2008).

### 2.12 Statistical analyses

SPSS Software (Version 8.2; SAS Institute, Cary, NC, USA) was used to conduct statistical analyses. These data were analyzed by one‐way analysis of variance (ANOVA). Comparison of means was performed by Duncan's multiple range tests and a *p *<* *.05 was considered statistically significant.

## 3 RESULTS AND DISCUSSION

### 3.1 Mold inhibitory potential of EOs in PDA medium

The results of the inhibitory potential of thyme and clove EOs against *A. flavus* growth and against aflatoxin B_1_ production by *A. flavus* in PDA medium are shown in Tables 1 and 2. As it can be inferred from Tables 1 and 2 that clove EO completely prevented fungal growth and showed inhibition of 100% at 200 ppm, while at the same concentration thyme EO showed inhibition of 86.7% and for complete inhibitory effect, 600 ppm of thyme EO was needed. Therefore, clove EO had stronger inhibitory potential than thyme EO for prevention of *A. flavus* growth. In a similar study, Mehrota et al. (2011) reported MIC of clove EO for *Staphylococcus aureus* as 0.25 μg/ml and in another study, Kumar et al. (2008) reported that the thyme EO completely inhibit the growth of *A. flavus* at concentration of 700 ppm.

**Table 1:** Effect of clove EO on growth and aflatoxin B_1_ production by *Aspergillus flavus* in PDA medium culture

EO concentration (ppm)	Colony diameter (mm)	Inhibition (%)	Aflatoxin B_1_ (μg/ml)	Inhibition (%)
0	30	—	3.75 ± 0.11	—
100	3.6 ± 0.8	100	ND	100
200	ND	100	ND	100
400	ND	100	ND	100
600	ND	100	ND	100
800	ND	100	ND	100

EO, essential oil; PDA, potato dextrose agar.

**Table 2:** Effect of thyme EO on growth and aflatoxin B_1_ production by *Aspergillus flavus* in PDA medium culture

EO concentration (ppm)	Colony diameter (mm)	Inhibition (%)	Aflatoxin B_1_ (μg/ml)	Inhibition (%)
0	30	—	3.75 ± 0.11	—
100	7.3 ± 1.2	75.8 ± 2.1^c^	0.24 ± 0.08	100
200	4 ± 1	86.7 ± 3.3^b^	ND	100
400	2 ± 1.7	93.3 ± 5.1^b^	ND	100
600	ND	100^a^	ND	100
800	ND	100^a^	ND	100

In each column different superscript letters indicate significant differences (*p < *.05).

EO, essential oil; PDA, potato dextrose agar.

The results in Tables 1 and 2 also showed that in PDA medium, the inhibitory potential of the two EOs against aflatoxin B_1_ production is greater than their inhibitory effect on *A. flavus* growth; for example, clove EO completely inhibited aflatoxin B_1_ production at 100 ppm, while double concentration of clove EO was needed to prevent *A. flavus* growth completely. The same trend was also observed for the thyme EO.

### 3.2 Inhibitory potential of EOs in PDB culture medium

Table 3 shows the inhibitory effects of EOs against *A. flavus* growth and aflatoxin B_1_ production by *A. flavus* in the PDB medium. As we expected, according to the obtained data, clove EO showed higher inhibitory effect than thyme EO. In this regard, clove EO showed inhibitory effect of 100% against *A. flavus* growth at concentration of 300 ppm, but for thyme EO the strongest inhibition effect (75%) was seen at concentration of 400 ppm. In a similar study, Razeghi‐Abyaneh et al. investigated and confirmed the antimicrobial effects of thymol and carvacrol in PDB medium against *A. flavus* growth.

**Table 3:** Effect of clove EO and thyme EO on *Aspergillus flavus* growth in PDB medium culture

EO concentration (ppm)	Clove EO		Thyme EO	
	Hyphae weight (mg)	Inhibition (%)	Hyphae weight (mg)	Inhibition (%)
0	737 ± 0.04	—	737 ± 0.04	—
100	107 ± 0.01	85.5 ± 1.2^c^	566 ± 0.04	23 ± 1.1^c^
200	50 ± 0.01	93.2 ± 0.1^b^	519 ± 0.02	29.6 ± 1^b^
300	ND	100^a^	185 ± 0.01	74.8 ± 0.8^a^
400	ND	100^a^	184 ± 0.01	75.1 ± 0.8^a^

In each column different superscript letters indicate significant differences (*p < *.05).

EO, essential oil; PDB, potato dextrose broth.

Comparison of Tables 1 and 2 data with Table 3 data shows that EOs in the PDB medium has less inhibitory potential against *A. flavus* growth than PDA medium. This phenomenon can be attributed to the following factors: (1) the presence of more nutrients in the PDB than PDA per unit weight (g) of culture medium; (2) the absence of agar in PDB culture medium and higher water availability of PDB than PDA medium; (3) presence of higher volume of dissolved oxygen in PDB medium than PDA medium; (4) higher incubation time of PDB medium (10 days) than that of PDA medium (3 days) and consequently inability of some inhibitory compounds to maintain their inhibitory effects when incubation time is prolonged. Accordingly, to achieve a correct comparison, the effect of time and amount of culture medium must be omitted for PDA and PDB culture medium. In this regard, all data related to PDA and PDB media were calculated on the basis of medium weight (g) and unit of time (day).

**Figure 1:**
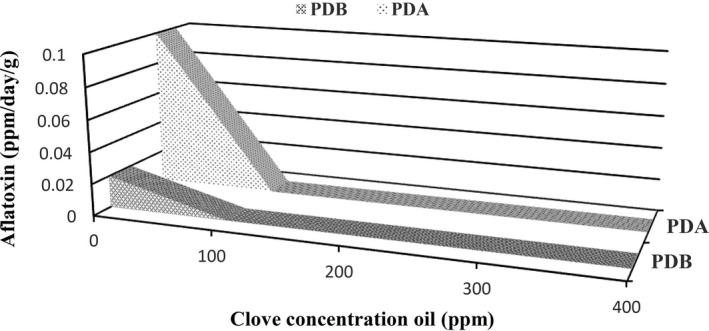
Comparison of inhibitory effects of clove EO on aflatoxin B_1_ production by *Aspergillus flavus* in PDA and PDB culture media. EO, essential oil; PDA, potato dextrose agar; PDB, potato dextrose broth

**Figure 2:**
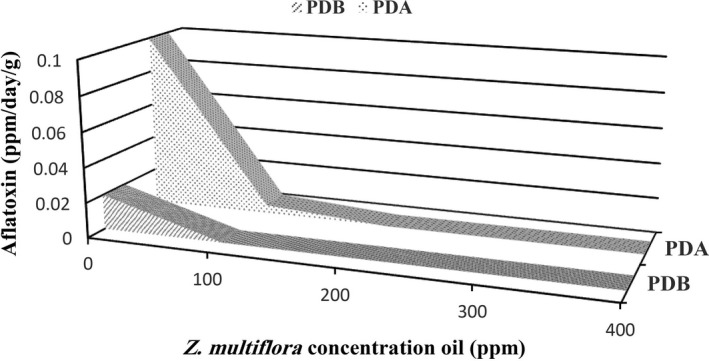
Comparison of inhibitory effects of thyme EO on aflatoxin B_1_ production by *Aspergillus flavus* in PDA and PDB culture media. EO, essential oil; PDA, potato dextrose agar; PDB, potato dextrose broth

The results of the inhibitory effects of the EOs against aflatoxin B_1_ production in both PDA and PDB media are shown in Figures 1 and 2. It was found that at the same time, the aflatoxin B_1_ production in PDA medium was higher than that of PDB medium, which was because of the freely movement of hyphae in the PDB culture medium and greater possibility to access nutrients during incubation. In the case of PDB medium, the hyphae remained at the growth phase for longer time and aflatoxin B_1_ production was postponed. Moreover, the pyruvate concentration per unit volume of the culture medium is a critical factor for aflatoxin B_1_ production by *A. flavus* and since in PDB medium the distribution of compounds is more extensive than PDA medium, the pyruvate concentration in PDB is less than that of PDA and consequently the aflatoxin B_1_ production was more restricted in PDB medium. Also, in PDA medium inhibitory effect of EOs was only limited to the location where surface of the hyphae was contacted to the solid media. While in the PDB medium, all of the essential oil had contact to the hyphae.

### 3.3 Inhibitory potential of EOs in Iranian white cheese as a food model

Results related to the inhibition effects of thyme and clove EOs in Iranian white cheese are presented in Table 4. By the end of the 40th day of storage, thyme EO showed the inhibitory potential of 86.81% at 200 ppm concentration against *A. flavus* growth and the highest inhibitory effect of thyme EO was recorded at 600 ppm, which was equal to 91.3% of inhibition. Moreover, because of high mold inhibitory potential of clove EO, which was evidenced in previous experiments*,* the essence showed 85.3%, 95%, and 100% of inhibition against *A. flavus* at concentrations of 50, 100, and 150 ppm, respectively. Similar to the results of the PDA and PDB medium, in Iranian white cheese the inhibitory effect of the EOs against *A. flavus* growth was higher than their inhibitory effect against aflatoxin B_1_ production. In the case of clove EO, 50 and 100 ppm of the essence could eradicate the aflatoxin B_1_ after 20 and 10 days of storage, respectively, and by introducing 150 ppm of the clove EO no aflatoxin were found during any time of storage.

**Table 4:** Effect of clove EO and thyme EO on *Aspergillus flavus* growth and aflatoxin B_1_ production by *Aspergillus flavus* in Iranian white cheese

Time (day)	Clove EO			Thyme EO		
	EO concentration (ppm)	*A. flavus* growth (cfu/ml × 10^4^)	Aflatoxin B_1_ concentration (ppm)	EO concentration (ppm)	*A. flavus* growth (cfu/ml × 10^4^)	Aflatoxin B_1_ concentration (ppm)
0	0	0	0	0	0	0
10		3.5 ± 0.7^c^	1,752 ± 127^d^		3.5 ± 0.7^c^	1,752 ± 127^d^
20		8.5 ± 0.7^b^	3,742 ± 226^c^		8.5 ± 0.7^b^	3,742 ± 226^c^
30		15 ± 1.4^a^	9,621 ± 42^a^		15 ± 1.4^a^	9,621 ± 42^a^
40		15.5 ± 2.1^a^	8,261 ± 304^b^		15.5 ± 2.1^a^	8,261 ± 304^b^
0	50	0	0	200	0	0
10		0.8 ± 0.8^d^	388 ± 49^a^		1.5 ± 0.57^b^	349 ± 21^c^
20		1.2 ± 0.5^c^	295 ± 84^b^		1.7 ± 0.5^b^	595 ± 42^b^
30		1.7 ± 0.5^b^	0		2 ± 0.81^ab^	593 ± 35^b^
40		2.3 ± 0.5^a^	0		3 ± 0.82^a^	1,020 ± 84^a^
0	100	0	0	400	0	0
10		0.3 ± 0.1^d^	192 ± 63^a^		1 ± 0.81^b^	218 ± 42^c^
20		0.5 ± 0.4^c^	0		1.3 ± 0.5^b^	331 ± 35^b^
30		0.8 ± 0.5^b^	0		1.5 ± 0.58^ab^	342 ± 21^b^
40		1.3 ± 0.5^a^	0		2 ± 0.8^a^	739 ± 63^a^
0	150	0	0	600	0	0
10		0	0		0.7 ± 0.5^c^	191 ± 35^d^
20		0	0		1.2 ± 0.1^b^	320 ± 21^c^
30		0	0		1.4 ± 0.6^ab^	482 ± 49^b^
40		0	0		1.7 ± 0.1^a^	584 ± 28^a^

In each column different superscript letters indicate significant differences (*p < *.05).

EO, essential oil.

Carvacrol, thymol, and eugenol are known as the most important polyphenolic compounds of thyme and clove EOs (López‐Malo, Alzamora, & Palou, 2005; Razzaghi‐Abyaneh et al., 2008), and lower levels of the EOs are needed to prevent the growth of microorganisms in vitro media compared to food samples (Bagamboula, Uyttendaele, & Debevere, 2004). Accordingly, the reduction of the inhibitory potential of the EOs against *A. flavus* growth and aflatoxin B_1_ production was ascribed to the created interactions between polyphenols and proteins of cheese (Ultee & Smid, 2001).

## 4 CONCLUSION

In this study it was found that EOs of thyme and clove have antifungal activity, so clove EO in PDA and PDB media and Iranian white cheese completely prevented the growth of *A. flavus* and aflatoxin B_1_ production. EO of thyme also with lower inhibitory potential compared with clove EO decreased *A. flavus* growth and aflatoxin B_1_ production. It was found that the inhibitory potential of each of the EOs in the PDB medium is more than PDA medium, plus deterrence potential of EOs of thyme and clove in a food environment was more than laboratory culture media. Finally, due to strong antifungal effects of EOs of thyme and clove, they can be used as a mold inhibitor in food. However, it is necessary that further studies to be done in the field of economic value and organoleptic effects of each of the EOs in the food.

## CONFLICT OF INTEREST

None declared.
